# Perioperative Validation of Two Handheld Glucometers in Dogs Under General Anesthesia: Analytical Robustness and Clinical Risk Assessment

**DOI:** 10.3390/ani16060993

**Published:** 2026-03-23

**Authors:** Catalina López, Valentina Hincapié, Jorge U. Carmona

**Affiliations:** 1 Grupo de Investigación Patología Clínica Veterinaria, Departamento de Salud Animal, Universidad de Caldas, Calle 65 No 26-10, Manizales 170004, Colombia; catalina.lopez@ucaldas.edu.co (C.L.); valentina.531714405@ucaldas.edu.co (V.H.); 2 Grupo de Investigación Terapia Regenerativa, Departamento de Salud Animal, Universidad de Caldas, Calle 65 No 26-10, Manizales 170004, Colombia

**Keywords:** perioperative monitoring, glucose measurement, dysglycemia, ISO 15197, point-of-care testing, intraoperative monitoring, clinical accuracy

## Abstract

Accurate perioperative glucose monitoring is essential in dogs undergoing general anesthesia, yet most validation studies of handheld glucometers have been performed under stable outpatient conditions. This prospective clinical validation study evaluated the analytical agreement, diagnostic performance, and ISO 15197 compliance of a human-calibrated (Accu-Chek) and a veterinary-specific (Centrivet GK) handheld glucometer compared with a laboratory spectrophotometric reference method in 34 anesthetized dogs (99 paired measurements per device). Linear mixed-effects modeling demonstrated significant method effects (*p* < 0.001), with the veterinary-specific device overestimating glucose concentrations relative to the reference method (β = 20.79 mg/dL; 95% CI: 8.08–33.50; *p* = 0.001), whereas the human-calibrated device did not differ significantly (β = 7.18 mg/dL; 95% CI: −5.53–19.89; *p* = 0.267). Bland–Altman analysis showed mean bias of 4.44 mg/dL (95% CI: 0.73–8.16) for the human-calibrated device and 22.72 mg/dL (95% CI: 18.22–27.21) for the veterinary-specific device. Passing–Bablok regression identified proportional bias only for the veterinary-specific device (slope 1.19; 95% CI: 1.01–1.34). ISO compliance was 69.7% and 39.4%, respectively. For hyperglycemia detection, AUC values were 0.9566 (95% CI: 0.8955–1.0000) and 0.9757 (95% CI: 0.9479–1.0000); for hypoglycemia, 0.8567 (95% CI: 0.7557–0.9578) and 0.7376 (95% CI: 0.6056–0.8697). In anesthetized dogs, the human-calibrated device demonstrated superior analytical robustness, whereas the veterinary-specific device showed greater bias and variability.

## Simple Summary

Maintaining normal blood sugar levels during anesthesia is important for the safety of dogs undergoing surgery. Both low and high blood sugar can occur during anesthesia and may affect the heart, brain, and recovery. Handheld blood glucose meters are commonly used in veterinary practice because they provide fast results using a small drop of blood. However, most previous studies evaluating these devices were performed in stable dogs, not during anesthesia, when blood flow and body temperature may change. This study compared two handheld glucose meters—one designed for humans and one designed for animals—with a laboratory method in dogs undergoing general anesthesia. The human-calibrated device showed smaller differences from the laboratory results and more consistent performance. The veterinary-specific device tended to overestimate glucose levels and showed greater variability. Although both devices were able to identify high blood sugar reliably, overall measurement accuracy was reduced during anesthesia. These findings suggest that handheld glucose meters should be carefully validated under real surgical conditions before being used for clinical decision-making in anesthetized dogs.

## 1. Introduction

Perioperative glucose homeostasis in dogs undergoing general anesthesia is dynamic and clinically relevant [1,2,3]. Both hypoglycemia and hyperglycemia may occur due to stress responses, anesthetic drugs, altered perfusion, intravenous fluid therapy, and the administration of alpha-2 adrenergic agonists [4,5,6,7,8,9,10]. Even transient dysglycemia may compromise cardiovascular stability, neurologic function, immune response, and postoperative recovery [11]. Accurate intraoperative glucose monitoring is therefore essential to avoid inappropriate therapeutic interventions that may compromise anesthetic safety. In particular, detection of hypoglycemia during general anesthesia is clinically critical, as inadequate recognition may compromise neurologic function and cardiovascular stability. Accurate perioperative glucose monitoring is therefore essential to support timely therapeutic decisions and maintain physiologic stability in anesthetized patients [6].

Handheld point-of-care glucometers (POCGs) are widely used in veterinary clinical practice because they provide rapid bedside results and require minimal blood volume. Both human-calibrated and veterinary-specific devices are commercially available and are frequently used in a variety of clinical contexts, including perioperative settings [12,13,14,15,16]. However, most validation studies in dogs have been conducted in outpatient or diabetic monitoring conditions, where physiological variables are relatively stable [1,14,16,17,18,19]. Although flash glucose monitoring systems (FGMSs) have been evaluated in perioperative and anesthetized dogs [20,21,22], these devices differ fundamentally from handheld electrochemical glucometers in measurement technology and sampling methodology [23]. Data specifically evaluating the analytical performance of traditional handheld glucometers in anesthetized dogs remain limited.

In contrast, the anesthetized patient represents a substantially different physiological environment. General anesthesia and commonly used agents, including alpha-2 adrenergic agonists, may induce peripheral vasoconstriction, alterations in cardiac output, and hemodynamic variability. Intraoperative fluid therapy and redistribution phenomena can modify hematocrit, while anesthetic-induced hypothermia is frequently observed in dogs undergoing surgery [24,25,26]. These factors have been shown to influence electrochemical glucose sensor performance, particularly through effects on peripheral perfusion, hematocrit-dependent strip calibration, and temperature-sensitive enzymatic reactions [27,28]. Moreover, repeated intraoperative measurements obtained from the same individual introduce within-subject correlation, which may bias traditional agreement analyses if not appropriately modeled [29].

International standards such as ISO 15197:2013 define analytical accuracy thresholds for glucose monitoring systems in human medicine [30]. Although ISO compliance has been evaluated in several veterinary studies conducted under outpatient or diabetic monitoring conditions [13,17,31], data assessing adherence to ISO criteria in anesthetized dogs remain limited. In this context, ISO 15197 is commonly used as a standardized analytical benchmark for comparative evaluation of portable glucometers, rather than as a regulatory criterion in veterinary medicine. Furthermore, beyond analytical agreement, the clinical diagnostic performance of traditional handheld glucometers for detecting hypo- and hyperglycemia during general anesthesia has not been extensively investigated.

Therefore, the objective of this study was to comprehensively evaluate the analytical agreement, clinical diagnostic performance, and ISO 15197 compliance of a human-calibrated and a veterinary-specific handheld glucometer compared with a laboratory spectrophotometric reference method in dogs undergoing general anesthesia. Particular emphasis was placed on the dynamic intraoperative setting, where repeated measurements and physiologic variability may influence device performance.

We hypothesized that the physiologic alterations associated with general anesthesia would result in measurable differences in analytical agreement and diagnostic performance between handheld glucometers and the laboratory reference method, and that compliance with ISO 15197 analytical accuracy criteria would be reduced under intraoperative conditions.

## 2. Materials and Methods

This prospective clinical validation study was conducted in client-owned dogs undergoing general anesthesia for elective or non-elective surgical procedures. The study protocol was approved by the Ethics Committee of the Universidad de Caldas (approval date: 9 February 2026), and written informed consent was obtained from all owners prior to enrollment. All procedures were conducted in accordance with institutional guidelines for the care and use of animals in research and in compliance with Colombian animal welfare legislation (Law 84 of 1989).

### 2.1. Study Design

Dogs of various breeds, ages, and body weights were enrolled consecutively during the study period. Inclusion criteria required that dogs undergo general anesthesia and have complete paired intraoperative glucose measurements obtained simultaneously by all three methods evaluated. Dogs with incomplete data were excluded from analysis.

Signalment variables including age, sex, body weight, surgical procedure, and use of alpha-2 adrenergic agonists were recorded. Body weight was recorded for all dogs as a continuous variable describing body size. Body condition score was not systematically recorded as part of the clinical dataset.

### 2.2. Anesthetic Protocol and Perioperative Management

General anesthesia was performed according to standard institutional protocols. Premedication, induction, and maintenance drugs were selected at the discretion of the attending anesthesiologist based on clinical indication. Use of alpha-2 agonists was documented. Intraoperative monitoring followed institutional standards, including cardiovascular and respiratory monitoring.

All procedures were performed in the surgical suites of the university veterinary hospital, where operating room temperature is maintained between 23 and 25 °C as part of routine environmental control during small animal anesthesia. In addition, patients routinely received active thermal support during anesthesia using warming blankets or similar devices as needed to maintain normothermia and minimize perioperative heat loss.

Glucose measurements were obtained at predefined intraoperative time points. These repeated measurements allowed assessment of dynamic changes during anesthesia.

### 2.3. Glucose Measurement Methods

At each time point, a single blood sample was collected and analyzed using three methods: (1) Laboratory spectrophotometric glucose determination using an automated chemistry analyzer (reference method), (BTS350 Chemistry Analyzer, BioSystems, Barcelona, Spain), (2) a human-calibrated handheld glucometer (Accu-Chek portable glucometer, Roche Diagnostics, Basel, Switzerland), and (3) a veterinary-specific handheld glucometer (Centrivet GK, ACON Laboratories Inc., San Diego, CA, USA).

Blood samples were obtained from peripheral venous access established for routine perioperative management, most commonly from the cephalic or saphenous vein.

Handheld measurements were performed immediately after sampling according to manufacturer instructions. The laboratory method served as the reference standard for all agreement and diagnostic analyses.

### 2.4. Statistical Analysis

All statistical analyses were performed using R statistical software (R Foundation for Statistical Computing, Vienna, Austria), version 4.5.2, within the RStudio environment. The following packages were used: lme4 and lmerTest for linear mixed-effects modeling, glmmTMB for generalized linear mixed modeling (sensitivity analyses), emmeans for estimated marginal means, mcr for Passing–Bablok regression, pROC for ROC analysis, dplyr and tidyr for data management, and ggplot2 for graphical visualization [32].

The dataset had a hierarchical structure with repeated intraoperative glucose measurements nested within dogs. To account for within-dog correlation, glucose concentration (mg/dL) was analyzed using a linear mixed-effects model. The dependent variable was glucose concentration, and fixed effects included measurement method, intraoperative time, sex, and the interaction between method and time. Dog identification was included as a random intercept to account for clustering of repeated measurements within individuals. The final model was specified as

glucose ~ method × time + sex + (1 | dog_id).

Model selection was guided by parsimony, Akaike Information Criterion (AIC), interpretability, and stability of parameter estimates. Alternative models including age, body weight, type of surgery, and use of alpha-2 agonists were explored but did not improve model fit or materially alter the primary effects; therefore, the most parsimonious stable model was retained for reporting [33,34].

Model assumptions were evaluated by inspection of residual plots and quantile–quantile plots. Statistical significance was assessed using Type III analysis of variance with Satterthwaite approximation for degrees of freedom [33,34]. Estimated marginal means were calculated to describe method-specific glucose trajectories across time.

Agreement between each handheld glucometer and the laboratory reference method was evaluated using Bland–Altman analysis [29]. Bias was calculated as the mean difference between test and reference measurements. Ninety-five percent limits of agreement were computed as bias ±1.96 times the standard deviation of the paired differences. Confidence intervals for bias and limits of agreement were calculated using standard parametric approximations to quantify the precision of agreement estimates [35]. Passing–Bablok regression was performed to evaluate constant and proportional bias between test and reference methods, with intercept and slope estimates and corresponding 95% confidence intervals calculated analytically [36].

Clinical diagnostic performance for detection of dysglycemia was evaluated using ROC analysis [37,38]. Dysglycemia was defined a priori using predefined laboratory reference thresholds, with hypoglycemia established as blood glucose ≤70 mg/dL and hyperglycemia as ≥150 mg/dL, based on accepted canine clinical reference intervals [39]. The area under the ROC curve (AUC) and corresponding 95% confidence intervals were calculated using DeLong’s method [40]. Discriminatory performance was interpreted using conventional criteria.

Analytical accuracy was further evaluated according to ISO 15197:2013 criteria [30]. For reference glucose concentrations below 100 mg/dL, measurements were considered compliant if the absolute difference between test and reference was ≤15 mg/dL. For reference concentrations equal to or greater than 100 mg/dL, compliance required agreement within ±15%. The proportion of paired measurements meeting ISO criteria was calculated for each device. Although ISO 15197 was originally developed for human self-monitoring systems, it was applied here as an objective analytical benchmark, consistent with previous veterinary validation studies.

No a priori sample size calculation was performed, as this study was designed as a prospective clinical validation study conducted under routine anesthetic conditions. The sample size was determined by the number of eligible dogs during the study period with complete paired measurements available [41]. Although 99 paired observations per device were available for agreement analyses, the number of independent experimental units was 34 dogs. Repeated intraoperative measurements increased precision for estimating device-related differences but do not increase the number of independent subjects. Ninety-nine paired observations are generally sufficient to estimate bias and limits of agreement with acceptable precision [42]. Confidence intervals were reported for agreement and diagnostic performance parameters to quantify estimation uncertainty. All tests were two-sided, and a *p*-value < 0.05 was considered statistically significant.

## 3. Results

### 3.1. Study Population and Descriptive Data

A total of 34 dogs were included in the study (Table 1), contributing glucose measurements obtained at predefined perioperative time points, including pre-anesthetic, intraoperative, and early recovery (prior to extubation) phases.

Each dog was scheduled to contribute three repeated measurements per method, yielding an expected total of 102 observations per device. After exclusion of time points lacking simultaneous measurements across all three methods, 99 paired observations per device relative to the laboratory reference method were available for agreement analyses.

Dogs represented a heterogeneous clinical population with respect to sex distribution, surgical indication, and use of alpha-2 adrenergic agonists. Age and body weight are summarized in Table 1 as mean ± SD and median (IQR), reflecting the variability expected in a general surgical population. The distribution of measurements across perioperative time points is detailed in Table 2. Sampling was balanced across pre-anesthetic, intraoperative, and recovery phases, with near-complete data acquisition at each stage.

### 3.2. Linear Mixed-Effects Model Results

Glucose concentration was analyzed using a linear mixed-effects model accounting for repeated measurements within dogs (Table 3). A significant main effect of measurement method was observed (Type III ANOVA, *p* < 0.001), as well as a significant effect of perioperative time (*p* < 0.001). Sex was also significantly associated with glucose concentration, with male dogs exhibiting lower glucose values compared with females (β = −20.96 mg/dL; 95% CI: −37.68 to −4.25; *p* = 0.015). The overall interaction between measurement method and time was not statistically significant (*p* = 0.764).

**Figure 1:**
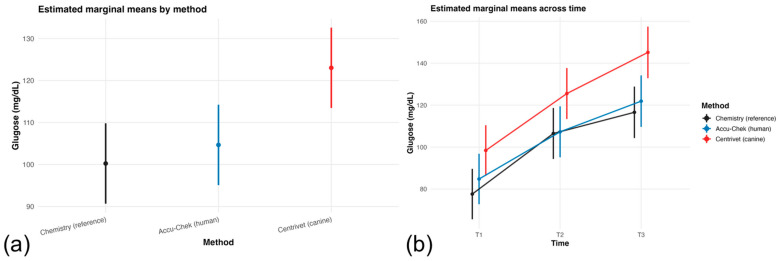
(**a**) Estimated marginal means (EMMs) of glucose concentration by measurement method derived from the linear mixed-effects model. The veterinary-specific device showed higher values than the laboratory reference method, whereas the human-calibrated device did not differ significantly. (**b**) EMMs of glucose concentration across perioperative time points. Glucose increased significantly over time, with no significant method × time interaction. Error bars represent 95% confidence intervals.

Compared with the laboratory reference method, the veterinary-specific device yielded significantly higher glucose concentrations (β = 20.79 mg/dL; 95% CI: 8.08 to 33.50; *p* = 0.001), whereas the human-calibrated device did not differ significantly from the reference method (β = 7.18 mg/dL; 95% CI: −5.53 to 19.89; *p* = 0.267). Estimated marginal means by measurement method are presented in Figure 1a, illustrating the higher overall glucose values obtained with the veterinary-specific device. Glucose concentrations increased progressively across perioperative time points, with significantly higher values observed at T2 (β = 28.88 mg/dL; *p* < 0.001) and T3 (β = 38.97 mg/dL; *p* < 0.001) compared with the pre-anesthetic baseline (T1). Longitudinal glucose trajectories for each method across time are shown in Figure 1b, demonstrating consistent upward trends during anesthesia and recovery.

A Gaussian generalized linear mixed model (GLMM) was fitted as a sensitivity analysis. Estimates for device-related effects were consistent in magnitude and direction with those obtained from the primary LMM, supporting robustness of the findings (Table S1).

### 3.3. Agreement Analysis

Bland–Altman analysis demonstrated distinct agreement patterns between each handheld glucometer and the laboratory reference method (Table 4).

**Figure 2:**
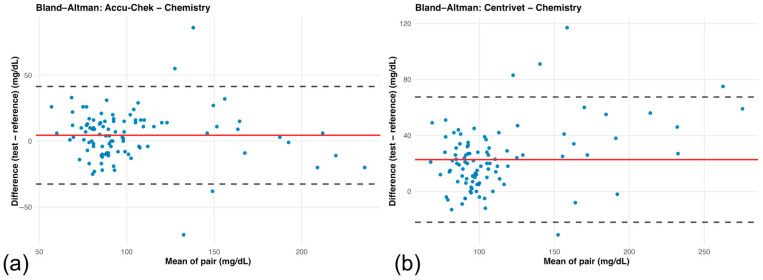
(**a**) Bland–Altman plot comparing the human-calibrated glucometer (Accu-Chek) with the laboratory reference method. Differences were calculated as (Accu-Chek—laboratory reference). The solid line represents the mean bias and dashed lines indicate the 95% limits of agreement (mean bias ± 1.96 SD). Positive values indicate higher readings by the handheld device. (**b**) Bland–Altman plot comparing the veterinary-specific glucometer (Centrivet GK) with the laboratory reference method. Differences were calculated as (Centrivet GK—laboratory reference). The solid line represents the mean bias and dashed lines the 95% limits of agreement. Positive values indicate higher measurements by the veterinary-specific device.

For the human-calibrated glucometer, the mean bias was 4.44 mg/dL (95% CI: 0.73 to 8.16), indicating a slight positive systematic difference relative to the laboratory method. The limits of agreement ranged from −32.52 to 41.41 mg/dL, reflecting moderate variability across the measurement range (Figure 2a). For the veterinary-specific glucometer, the mean bias was substantially higher at 22.72 mg/dL (95% CI: 18.22 to 27.21), demonstrating consistent positive bias. Limits of agreement were wider (−21.99 to 67.43 mg/dL), indicating greater dispersion of differences compared with the human-calibrated device (Figure 2b).

Visual inspection of the Bland–Altman plots suggested greater dispersion of differences at higher mean glucose concentrations for the veterinary-specific device, consistent with the proportional bias identified in Passing–Bablok regression.

### 3.4. Passing–Bablok Regression

Passing–Bablok regression analysis further characterized agreement between each handheld glucometer and the laboratory reference method (Table 5). For the human-calibrated device (Accu-Chek), the slope was 0.99 (95% CI: 0.88 to 1.11), indicating no significant proportional bias, as the confidence interval included 1.0. The intercept was 6.68 mg/dL (95% CI: −5.30 to 17.91), with the confidence interval including 0, suggesting absence of significant constant bias.

In contrast, the veterinary-specific device (Centrivet GK) demonstrated a slope of 1.19 (95% CI: 1.01 to 1.34), consistent with significant proportional bias, as the confidence interval did not include 1.0. The intercept was 2.57 mg/dL (95% CI: −13.18 to 19.93), indicating no significant constant bias. These findings suggest that differences between the veterinary-specific device and the laboratory method increased at higher glucose concentrations.

### 3.5. ISO 15197 Analytical Accuracy

Compliance with ISO 15197:2013 analytical accuracy criteria differed substantially between devices (Table 6). The human-calibrated glucometer met ISO accuracy requirements in 69.7% of paired measurements, whereas the veterinary-specific device met criteria in 39.4% of paired measurements. Accordingly, neither device achieved the ISO requirement of ≥95% of measurements within predefined analytical limits.

**Figure 3:**
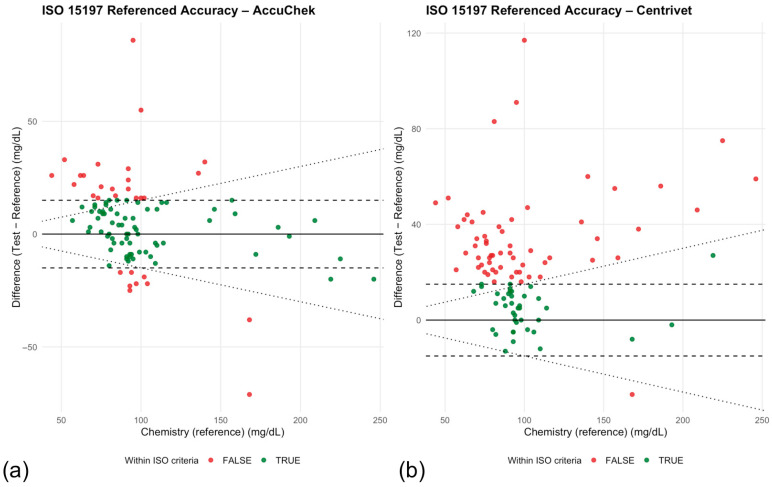
(**a**) Human-calibrated glucometer (Accu-Chek). (**b**) Veterinary-specific glucometer (Centrivet GK). Differences were calculated as (test device − laboratory reference). Points are color-coded according to compliance status (green = within ISO criteria; red = outside ISO criteria). Dotted lines represent ISO 15197:2013 allowable analytical error limits (±15 mg/dL for reference glucose concentrations <100 mg/dL and ±15% for concentrations ≥100 mg/dL). The horizontal solid line indicates zero difference.

Graphical representation of ISO compliance relative to reference glucose concentrations is shown in Figure 3a,b. For the human-calibrated device (Figure 3a), most measurements falling outside ISO criteria were distributed across both low and high glucose ranges, with no clear concentration-dependent pattern. In contrast, the veterinary-specific device (Figure 3b) demonstrated a higher proportion of non-compliant measurements, particularly at higher glucose concentrations, consistent with the proportional bias observed in Passing–Bablok regression.

### 3.6. Diagnostic Performance

#### 3.6.1. ROC Analysis

Diagnostic performance for detection of dysglycemia was evaluated using ROC analysis with the laboratory reference method as the gold standard (Table 7).

**Figure 4:**
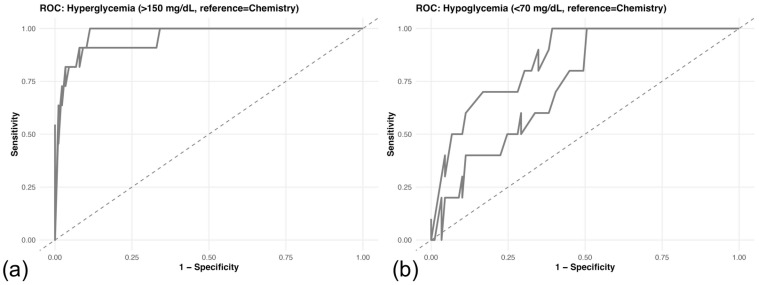
Receiver operating characteristic (ROC) analysis for detection of dysglycemia relative to the laboratory reference method. (**a**) Hyperglycemia (>150 mg/dL). (**b**) Hypoglycemia (<70 mg/dL). AUC values with 95% confidence intervals were calculated using DeLong’s method. For hyperglycemia, AUCs were 0.9566 for the human-calibrated glucometer and 0.9757 for the veterinary-specific glucometer. For hypoglycemia, AUCs were 0.8567 and 0.7376, respectively. The diagonal dashed line indicates no discrimination (AUC = 0.5); curves closer to the upper left corner reflect better diagnostic performance.

For detection of hyperglycemia (>150 mg/dL; 11 events), both devices demonstrated excellent discriminatory ability. The human-calibrated glucometer achieved an AUC of 0.9566 (95% CI: 0.8955–1.0000), and the veterinary-specific device achieved an AUC of 0.9757 (95% CI: 0.9479–1.0000) (Figure 4a).

For detection of hypoglycemia (<70 mg/dL; 10 events), performance was lower. The human-calibrated device showed good discrimination (AUC = 0.8567, 95% CI: 0.7557–0.9578), whereas the veterinary-specific device demonstrated moderate discrimination (AUC = 0.7376, 95% CI: 0.6056–0.8697) (Figure 4b). Given the limited number of dysglycemic events, confidence intervals should be interpreted cautiously.

#### 3.6.2. Clinical Error Grid Results

**Figure 5:**
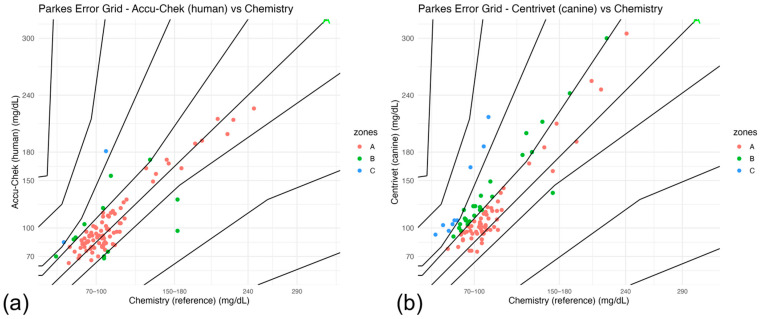
Parkes (Consensus) Error Grid analysis relative to the laboratory reference method. (**a**) Human-calibrated glucometer (Accu-Chek). (**b**) Veterinary-specific glucometer (Centrivet GK). Each point represents a paired perioperative glucose measurement. The diagonal line indicates perfect agreement. The grid is divided into clinical risk zones (A–D), reflecting the potential impact of measurement error on therapeutic decisions. Zones A and B indicate clinically acceptable results, whereas Zones C–D represent increasing clinical risk.

Parkes (Consensus) error grid analysis was performed to evaluate the potential clinical impact of measurement error relative to the laboratory reference method (chemistry). The distribution of paired measurements across clinical risk zones (A–E) is summarized in Table 8, and graphical representations are shown in Figure 5a,b.

For the human-calibrated glucometer (Accu-Chek), the majority of measurements fell within Zones A and B, indicating clinically acceptable agreement in most cases. Only a small proportion of paired observations were located in Zones C–E, suggesting limited risk of clinically significant misclassification (Figure 5a).

In contrast, the veterinary-specific device (Centrivet GK) showed a lower proportion of measurements in Zone A and a greater distribution across Zones B and C (Figure 5b). Although most observations for both devices remained within clinically acceptable Zones A+B, deviations beyond these zones were more frequent for the veterinary-specific device (9.1% vs. 2.0%). Fisher’s exact test indicated a strong trend toward increased clinically relevant deviations for Centrivet GK (OR = 4.85, *p* = 0.058), although statistical significance was not reached. These findings are consistent with the proportional bias and wider limits of agreement identified in Bland–Altman analysis and collectively suggest comparatively reduced clinical reliability under perioperative conditions.

## 4. Discussion

This study provides a comprehensive evaluation of handheld glucometer performance in dogs undergoing general anesthesia, integrating analytical agreement, regression-based bias assessment, ISO 15197 compliance, diagnostic discrimination, and clinical risk analysis [30,43]. The findings indicate that device performance under dynamic intraoperative conditions differs from that reported in more stable outpatient monitoring environments [13,44]. Although both devices exhibited strong discriminatory capacity for hyperglycemia, analytical agreement and ISO compliance were reduced, particularly for the veterinary-specific device, highlighting the important distinction between diagnostic discrimination and analytical interchangeability [29,41].

The linear mixed-effects model revealed systematic differences between devices. Importantly, the use of repeated intraoperative measurements represents a methodological strength of the study, allowing characterization of dynamic glucose changes during anesthesia while accounting for within-subject variability. The veterinary-specific glucometer consistently overestimated glucose concentrations relative to the laboratory reference method, whereas the human-calibrated device demonstrated smaller and statistically non-significant differences. The absence of a significant method-by-time interaction indicates that device-related differences were stable across perioperative stages rather than confined to specific intraoperative phases. Mixed-effects modeling appropriately accounted for repeated measurements within individual dogs, strengthening the reliability of the reported estimates.

Agreement analysis further clarified device behavior. Bland–Altman results demonstrated wider limits of agreement for the veterinary-specific device, indicating greater variability and reduced interchangeability with the laboratory reference method. Passing–Bablok regression confirmed proportional bias for this device, with increasing divergence at higher glucose concentrations [36]. Similar patterns of systematic differences between portable glucometers and reference methods have been described in dog studies, where different devices showed variable agreement and analytical performance compared with laboratory assays [13,14,45,46,47]. However, most of those studies were conducted in non-anesthetized outpatient or diabetic populations, and the magnitude of bias observed in the present intraoperative setting appears more pronounced. To the authors’ knowledge, no prior peer-reviewed studies have specifically evaluated the analytical performance of this veterinary-specific handheld glucometer in dogs, further emphasizing the importance of independent validation under perioperative conditions.

Interestingly, when the same veterinary-specific device was evaluated under field conditions in dairy cattle, limited analytical agreement with laboratory reference methods was also reported, including evidence of proportional bias and wide limits of agreement [48]. Although direct cross-species comparisons are not appropriate due to physiological and sampling differences, these findings collectively suggest that device performance may be context-dependent and influenced by species-specific and environmental conditions. These complementary findings suggest that systematic overestimation may be amplified under anesthetic conditions characterized by altered perfusion, temperature variation, and potential hematocrit fluctuations, thereby reducing analytical precision in this dynamic clinical context [25]. However, hematocrit and objective perfusion metrics were not directly measured or modeled in the present study; therefore, these mechanisms should be interpreted as biologically plausible hypotheses rather than directly evaluated variables.

From a diagnostic perspective, both devices performed well in identifying hyperglycemia, with AUC values approaching 1.0. However, performance for hypoglycemia detection was lower and associated with wider confidence intervals, particularly for the veterinary-specific device. The broader confidence intervals likely reflect the limited number of hypoglycemic events, as estimation precision in ROC analysis is directly influenced by event frequency [37,40]. Therefore, diagnostic performance for hypoglycemia should be interpreted as exploratory and hypothesis-generating rather than definitive, and confirmation in larger datasets will be required.

Despite this statistical limitation, the clinical implications remain important. Detection of hypoglycemia during general anesthesia is critical, as inadequate recognition may compromise neurologic function and cardiovascular stability [25,49]. Devices exhibiting greater analytical variability in the lower glucose range may therefore present increased clinical risk, particularly in dynamic perioperative settings. Importantly, excellent ROC performance did not translate into compliance with ISO 15197 analytical accuracy criteria. ISO standards assess absolute measurement accuracy relative to predefined error thresholds [30], whereas ROC analysis evaluates discriminatory ability across a range of values [37]. A device may therefore reliably classify hyperglycemia while still demonstrating clinically relevant measurement bias. In the present study, neither device achieved the ISO requirement of ≥95% compliant measurements under intraoperative conditions. The human-calibrated device met ISO criteria in approximately two-thirds of measurements, whereas the veterinary-specific device demonstrated substantially lower compliance. These findings suggest that manufacturer-reported accuracy metrics derived from controlled or outpatient conditions may not fully reflect performance during anesthesia.

ISO 15197 was originally developed for human self-monitoring systems and was used in this study as a standardized analytical benchmark rather than a strict regulatory pass–fail criterion in veterinary anesthesia. Because intraoperative physiology differs from stable outpatient conditions, reduced compliance with ISO thresholds likely reflects the dynamic metabolic environment during anesthesia rather than an inherent lack of clinical utility of the devices.

Error grid analysis provided further insight into the clinical implications of measurement error. The human-calibrated device demonstrated a high proportion of measurements within Zones A and B, indicating predominantly acceptable clinical agreement. In contrast, the veterinary-specific device showed a greater proportion of measurements in Zone C, consistent with the proportional bias and wider limits of agreement observed in analytical analyses. The Parkes (Consensus) error grid categorizes measurement deviations according to their potential impact on clinical decision-making, with Zones A and B considered clinically acceptable and Zones C–E representing increasing levels of clinical risk [50,51]. Although most measurements for both devices remained within clinically acceptable zones, the increased dispersion for the veterinary-specific device suggests a higher likelihood of clinically meaningful misclassification under certain circumstances.

Previous studies have evaluated flash glucose monitoring systems (FGMSs) in dogs undergoing anesthesia or perioperative management, reporting acceptable agreement with reference methods in selected clinical settings [20,21]. However, FGMS devices measure interstitial glucose rather than whole-blood glucose and are subject to physiological lag time between plasma and interstitial compartments. In contrast, handheld electrochemical glucometers directly analyze capillary or venous blood samples, thereby reflecting immediate circulating glucose concentrations [16]. Differences in sampling matrix and sensor technology may therefore influence analytical behavior under dynamic intraoperative conditions. While FGMSs have demonstrated clinical feasibility in anesthetized dogs, their analytical performance characteristics are not directly comparable to those of handheld point-of-care blood glucometers. The present findings suggest that even direct blood-based electrochemical devices may exhibit increased variability under anesthesia, emphasizing that device performance must be interpreted within the specific technological and physiological context in which monitoring occurs [20,21].

The intraoperative environment presents unique physiological challenges that may contribute to these discrepancies [24,25]. General anesthesia is associated with altered peripheral perfusion, vasoconstriction induced by alpha-2 adrenergic agonists, hemodynamic variability, and temperature fluctuations [26]. Additionally, intraoperative fluid shifts may influence hematocrit and plasma distribution dynamics, potentially affecting electrochemical sensor performance. These factors may amplify measurement variability in ways not captured in traditional validation studies conducted under physiologically stable conditions. Consequently, intraoperative validation represents a more stringent test of device performance [11].

These mechanisms are plausible contributors; however, hematocrit and objective perfusion metrics were not directly modeled in this study, and causal attribution cannot be established. Future perioperative validation studies should incorporate hematocrit and hemodynamic variables to quantify their contribution to device-specific bias.

This study has several strengths. It was conducted in a real-world surgical population, incorporated repeated perioperative measurements, and employed a multidimensional analytical framework combining mixed modeling, agreement analysis, proportional bias assessment, ISO benchmarking [30], ROC analysis [41], and clinical error grid evaluation [43]. This comprehensive approach allows nuanced interpretation of both analytical and clinical performance characteristics.

Several limitations warrant consideration. The number of hypoglycemic events was limited, resulting in wider confidence intervals for diagnostic performance estimates in this range [37,40]. Hematocrit was not directly modeled as a covariate, and its potential influence on device accuracy merits further investigation [27,43,47]. Additionally, results may not be generalizable to critically ill or severely hemodynamically unstable patients, in whom perfusion abnormalities may be more pronounced.

Although 99 paired observations per device were available, the number of independent experimental units was 34 dogs. Repeated measurements increased precision for estimating average device-related differences but do not increase the number of independent subjects. In addition, hypoglycemic and hyperglycemic events were infrequent (10 and 11 events, respectively), leading to wider confidence intervals for ROC-derived estimates; therefore, diagnostic performance results (particularly for hypoglycemia) should be interpreted as exploratory.

Taken together, these findings indicate that intraoperative conditions represent a more demanding analytical environment for point-of-care glucose monitoring. Physiological alterations inherent to general anesthesia may amplify measurement variability and device-specific bias, underscoring the importance of validating handheld glucometers within the exact clinical context in which they are intended to be used.

## 5. Conclusions

In conclusion, handheld glucometers showed variable analytical performance in dogs undergoing general anesthesia. The human-calibrated device demonstrated comparatively better agreement with the laboratory reference method, whereas the veterinary-specific device showed greater bias and variability. These findings highlight the need for cautious interpretation of perioperative glucose measurements and for context-specific validation of point-of-care glucose monitoring systems in anesthetized dogs.

## Figures and Tables

**Table 1:** Demographic and Clinical Characteristics of the Study Population (n = 34).

Variable	Value
**Number of dogs**	34
**Age (months)**	
-Mean ± SD	61.6 ± 46.5
-Median (IQR)	61.5 (77.3)
-Range	5–168
**Body weight (kg)**	
-Mean ± SD	21.3 ± 13.1
-Median (IQR)	16.8 (14.8)
-Range	5.4–68
**Sex, n (%)**	
-Female	18 (52.9%)
-Male	16 (47.1%)
**Breed category, n (%)**	
-Mixed breed	6 (17.6%)
-Purebred (various breeds)	28 (82.4%)
**Alpha-2 agonist use, n (%)**	
-Xylazine	24 (70.6%)
-Dexmedetomidine	3 (8.8%)
-None	7 (20.6%)
**Surgical category, n (%)**	
-Reproductive	15 (44.1%)
-Soft tissue	17 (50.0%)
-Orthopedic	2 (5.9%)

Values are reported as mean ± SD, median (IQR), range, or n (%).

**Table 2:** Distribution of Glucose Measurements Across Perioperative Time Points.

Time Point	Clinical Phase	Total Measurements (All Methods)	Complete Paired Observations (per Device)
T1	Pre-anesthetic	102	34
T2	Intraoperative	99	33
T3	Recovery (pre-extubation)	96	32
**Total**	**—**	**297**	**99**

T, Time.

**Table 3:** Linear Mixed-Effects Model for Perioperative Glucose Concentration in Dogs.

Fixed Effect	Estimate (β, mg/dL)	SE	t-Value	df	95% CI	*p*-Value
**Intercept**	88.10	7.09	12.43	81.6	74.00 to 102.21	<0.001
Accu-Chek (human)	7.18	6.45	1.11	263.2	−5.53 to 19.89	0.267
Centrivet GK (canine)	20.79	6.45	3.22	263.2	8.08 to 33.50	0.001
Time T2	28.88	6.51	4.44	263.4	16.06 to 41.70	<0.001
Time T3	38.97	6.57	5.93	263.7	26.03 to 51.90	<0.001
Male (vs. Female)	−20.96	8.23	−2.55	34.2	−37.68 to −4.25	0.015
Accu-Chek × T2	−6.39	9.20	−0.69	263.2	−24.50 to 11.72	0.488
Centrivet GK × T2	−1.76	9.20	−0.19	263.2	−19.87 to 16.35	0.848
Accu-Chek × T3	−1.86	9.27	−0.20	263.2	−20.12 to 16.39	0.841
Centrivet GK × T3	7.77	9.27	0.84	263.2	−10.48 to 26.02	0.403

Reference categories: Laboratory method, pre-anesthetic time point (T1), and female dogs. The estimate for the Accu-Chek device (β = 7.18 mg/dL) represents the mean difference relative to the laboratory method under these reference conditions. Thus, at T1 in female dogs, the human-calibrated glucometer measured on average 7.18 mg/dL higher glucose concentrations than the laboratory method, although this difference was not statistically significant. Acronyms as in Table 2.

**Table 4:** Bland–Altman Agreement Statistics.

Device	Mean Bias (mg/dL)	95% CI of Bias	Lower LoA	Upper LoA
Accu-Chek	4.44	0.73 to 8.16	−32.52	41.41
Centrivet GK	22.72	18.22 to 27.21	−21.99	67.43

Differences were calculated as test device minus laboratory reference method. A positive mean bias indicates that the handheld device measured higher glucose concentrations than the laboratory method on average. Limits of agreement (LoA) represent the mean bias ± 1.96 standard deviations of the paired differences and define the interval within which approximately 95% of individual differences are expected to lie.

**Table 5:** Passing–Bablok regression analysis comparing each handheld glucometer with the laboratory reference method.

Device	Intercept (95% CI)	Slope (95% CI)	Interpretation
Accu-Chek	6.68 (−5.30 to 17.91)	0.99 (0.88 to 1.11)	No constant or proportional bias
Centrivet GK	2.57 (−13.18 to 19.93)	1.19 (1.01 to 1.34)	Significant proportional bias

Passing–Bablok regression was performed comparing each handheld device against the laboratory reference method. An intercept confidence interval including 0 indicates absence of significant constant bias. A slope confidence interval including 1.0 indicates absence of significant proportional bias. Confidence intervals not including these reference values suggest statistically significant bias.

**Table 6:** ISO 15197 analytical accuracy compliance of handheld glucometers relative to the laboratory reference method.

Device	n	Within ISO (%)	Outside ISO (%)
Accu-Chek (human)	99	69.7	30.3
Centrivet GK (canine)	99	39.4	60.6

ISO 15197:2013 requires that at least 95% of measurements fall within ±15 mg/dL of the reference value for glucose concentrations <100 mg/dL and within ±15% for concentrations ≥100 mg/dL. Percentages shown represent the proportion of paired measurements meeting these criteria relative to the laboratory reference method.

**Table 7:** Diagnostic performance (area under the ROC curve with 95% confidence intervals) for detection of dysglycemia relative to the laboratory reference method.

Condition	Events (n)	Accu-Chek AUC (95% CI)	Centrivet GK AUC (95% CI)
Hypoglycemia (<70 mg/dL)	10	0.8567 (0.7557–0.9578)	0.7376 (0.6056–0.8697)
Hyperglycemia (>150 mg/dL)	11	0.9566 (0.8955–1.0000)	0.9757 (0.9479–1.0000)

Area under the curve (AUC) values and 95% confidence intervals were calculated using DeLong’s method. An AUC of 0.5 indicates no discriminatory ability, whereas values approaching 1.0 indicate excellent diagnostic performance. Confidence intervals reflect uncertainty related to the limited number of dysglycemic events.

**Table 8:** Parkes (Consensus) Error Grid distribution relative to the laboratory reference method.

Device	Zone A n (%)	Zone B n (%)	Zone C n (%)	Zone D n (%)	Zone E n (%)	Zones A+B n (%)
Accu-Chek (human)	85 (85.9)	12 (12.1)	2 (2.0)	0 (0)	0 (0)	97 (98.0)
Centrivet GK (canine)	63 (63.6)	27 (27.3)	9 (9.1)	0 (0)	0 (0)	90 (90.9)

Parkes (Consensus) error grid classification comparing each glucometer with the laboratory reference method. Zone A represents clinically accurate results leading to correct treatment decisions. Zone B includes measurements with minimal clinical impact. Zones C–E represent increasing degrees of potential clinical risk. Zones A+B reflect overall clinically acceptable performance.

## Data Availability

The raw data supporting the conclusions of this article will be made available by the authors on request.

## Supplementary Materials

The following supporting information can be downloaded at: https://www.mdpi.com/article/10.3390/ani16060993/s1, Table S1. Generalized Linear Mixed Model (GLMM) Sensitivity Analysis for Glucose Concentration.

## Abbreviations

The following abbreviations are used in this manuscript:

POCG	Point-of-Care Glucometer
FGMS	Flash Glucose Monitoring System
ASVCP	American Society for Veterinary Clinical Pathology
LMM	Linear Mixed-Effects Model
AIC	Akaike Information Criterion
ROC	Receiver Operating Characteristic
AUC	Area Under the Curve
CI	Confidence Interval
SE	Standard Error
SD	Standard Deviation
IQR	Interquartile Range
LoA	Limits of Agreement
ISO	International Organization for Standardization
