# Supplementary material for: Perioperative Validation of Two Handheld Glucometers in Dogs Under General Anesthesia: Analytical Robustness and Clinical Risk Assessment
Source: Animals (Basel). 2026 Mar 23;16(6):993. doi: 10.3390/ani16060993 (PMC13024211; doi:10.3390/ani16060993)
Supplement: Supplementary file 1 [file animals-16-00993-s001.zip › Table S1.pdf]

Table S1. Generalized Linear Mixed Model (GLMM) Sensitivity Analysis for Glucose Concentration.

Fixed Effect	Estimate (β, mg/dL)	SE	z-value	95% CI	p-value
Intercept	88.10	7.09	12.43	74.21 to 102.00	<0.001
**Accu-Chek (human)**	7.18	6.45	1.11	−5.47 to 19.83	0.266
**Centrivet (canine)**	20.79	6.45	3.22	8.14 to 33.45	0.001
**Time T2**	28.88	6.51	4.44	16.12 to 41.64	<0.001
**Time T3**	38.97	6.57	5.93	26.09 to 51.84	<0.001
**Male (vs Female)**	−20.96	8.23	−2.55	−37.09 to −4.84	0.011
**Accu-Chek × T2**	−6.39	9.20	−0.69	−24.42 to 11.64	0.487
**Centrivet × T2**	−1.76	9.20	−0.19	−19.79 to 16.26	0.848
**Accu-Chek × T3**	−1.86	9.27	−0.20	−20.03 to 16.30	0.841
**Centrivet × T3**	7.77	9.27	0.84	−10.40 to 25.94	0.402

Gaussian generalized linear mixed model including measurement method, perioperative time, sex, and the method-by-time interaction as fixed effects, with dog identification included as a random intercept to account for repeated measurements within individuals. Estimates were consistent in magnitude and direction with those obtained from the primary linear mixed-effects model, supporting robustness of device-related effects. Reference categories were the laboratory spectrophotometric method, Time 1 (pre-anesthetic), and female sex. The intercept represents the estimated mean glucose concentration under these reference conditions. Device coefficients represent the mean difference in glucose concentration relative to the laboratory method at Time 1 in female dogs.
